# Analysis of risk factors and prognosis in differentiated thyroid cancer with focus on minimal extrathyroidal extension

**DOI:** 10.1186/s12902-021-00826-2

**Published:** 2021-08-10

**Authors:** Manuel Weber, Ina Binse, Karin Oebbecke, Tim Brandenburg, Ken Herrmann, Sarah Theurer, Frank Weber, Ann-Kathrin Ehrlich, Kurt Werner Schmid, Dagmar Führer-Sakel, Irfan Vardarli, Wolfgang P. Fendler, Elena Gilman, Rainer Görges

**Affiliations:** 1 Clinic for Nuclear Medicine, University Hospital Essen, Essen, Germany; 2 University of Duisburg-Essen and German Cancer Consortium (DKTK)-University Hospital, Hufelandstraße 55, 45147 Essen, Germany; 3 Department of Endocrinology, Diabetes and Metabolism, University Hospital Essen, University of Duisburg-Essen, Essen, Germany; 4 Institute of Pathology, University Hospital Essen, Essen, Germany; 5 Department of Surgery, Section Endocrine Surgery, Essen University Hospital, Essen, Germany; 6 Department of Medicine I, Klinikum Vest GmbH, Knappschaftskrankenhaus Recklinghausen, Academic Teaching Hospital, Ruhr-University Bochum, Recklinghausen, Germany; 7 Gilman Biometrics, Elena Gilman, Leipziger Strasse 18, 50858 Köln, Germany

**Keywords:** Differentiated thyroid cancer, DTC, Minimal extrathyroidal extension, AJCC/TNM classification

## Abstract

**Aims:**

In contrast to all prior AJCC/TNM classifications for differentiated thyroid cancer (DTC) the 8th edition does not take minimal extrathyroidal extension (M-ETE) into consideration for local tumor staging. We therefore aimed to retrospectively assess the specific impact of M-ETE on the outcome of M-ETE patients treated in our clinic.

**Methods:**

DTC patients with M-ETE and a follow-up time of ≥ 5 years were included and matched with an identical number of patients without M-ETE, but with equal histopathological tumor subtype and size. The frequency of initially metastatic disease among groups was compared using Fisher’s exact test, the recurrence rate by virtue of log-rank test. Fisher’s exact test and multivariate analysis were used to account for the presence of confounding risk factors.

**Results:**

One hundred sixty patients (80 matching pairs) were eligible. With other confounding risk factors being equal, the prevalence of N1-/M1-disease at initial diagnosis was comparable among groups (M-ETE: 42.5 %; no M-ETE: 32.5 %; *p* = 0.25). No differences with regard to the recurrence rate were shown. However, M-ETE patients were treated with external beam radiation therapy more often (16.3 % vs. 1.3 %; *p* = 0.004) and received higher median cumulative activities of ^131^I (10.0 vs. 8.0 GBq; *p* < 0.001).

**Discussion:**

Although having played a pivotal role for local tumor staging of DTC for decades M-ETE did not increase the risk for metastases at initial diagnosis and the recurrence rate in our cohort. Patients with M-ETE had undergone intensified treatment, which entails a possible confounding factor that warrants further investigation in randomized controlled trials.

## Background

With 53,990 new cases in the USA thyroid cancer is among the most prevalent cancers. Women are affected disproportionately and make up 40,900 of new cases [1]. Over the last decades the incidence of thyroid cancer has increased steadily [2]. A steep rise has been observed especially in papillary thyroid carcinoma (PTC) including microcarcinomas [3]. Explanations range from potential overdiagnosis of clinically inapparent carcinomas due to the widespread applications of diagnostic procedures to higher iodine intake [4–6]. However, this rise in incidence has neither been observed for anaplastic thyroid cancer nor follicular thyroid carcinoma (FTC) [7].

Despite the increasing incidence of DTC disease-specific survival rates remain excellent [8]. These changes in prevalence, age of onset and tumor size call for a critical re-evaluation of current therapeutic and diagnostic guidelines to allow for accurate risk stratification and optimal treatment. Consequently, with the advent of the 2017 American Joint Committee on Cancer (AJCC) TNM classification of thyroid cancer a few changes have been implemented:

In contrast to previous editions tumors showing minimal extrathyroidal extension (M-ETE) are no longer categorized as pT3 (6th and 7th edition) or pT4 (5th edition), but according to tumor size with the only exception being macroscopic infiltration of the extrathyroidal tissue. This caused further downstaging in many patients and is still a topic of debate leading to the proposal of a revision that takes M-ETE into account [9–11]. This revision aims at establishing a standardized reporting framework for M-ETE to assess its independent impact on patient prognosis in the future, since prior studies have shown conflicting evidence [12–16].

Therefore, in this study we examined minimal extrathyroidal extension in DTC as an independent risk factor for the presence of metastases at initial diagnosis and the risk of recurrence.

## Methods

### Patient selection

Eighty consecutive patients with M-ETE subgroup had to meet the following inclusion criteria:

- Treatment at the Clinic for Nuclear Medicine, University Hospital Essen.
- Follow up time of ≥ 5 years.
- Histopathologically confirmed PTC/FTC.
- Comprehensive staging according to TNM criteria.
- Primary tumor with minimal extrathyroidal extension, defined as extension to the thyroid capsule, perithyroidal soft tissue, or sternothyroid muscle.

### Matching process

To analyze the impact of M-ETE, propensity score matching was performed by selecting an equal number of consecutive patients with identical histopathological subtype and tumor size (deviation of ≤ 1 cm), but primary confined to the thyroid gland (TCT).

### Data acquisition

Besides generic data (e.g. name, age, sex, age at initial diagnosis, histopathological entity) the following data were collected:

- Staging according to TNM classification including tumor size.
- Course of the disease (especially time and location of recurrence).
- Unifocal vs. multifocal tumor lesions.
- Extrathyroidal extension.

Additionally, information about other risk factors not included into the matching process, such as sex, age > 55, incomplete tumor resection, multifocal tumor lesions and (for the analysis of the recurrence rate) prior treatment (radioactive iodine therapy (RAIT), external beam radiation therapy (EBRT)) was collected and their influence on the results analyzed [17–19]. The relevance of other suspected risk factors, such as BRAF mutation status and resection extent of primary operation has yet to be established and was beyond the scope of this publication [20].

### Statistical analysis

Statistical analysis was performed using IBM SPSS Statistics for Windows, version 25 (IBM Corp., Armonk, N.Y., USA), We employed a Chi²-Test and Fisher’s exact test to test for significant differences among groups of nominal variables. Interval scaled variables were compared using a t-Test for independent samples. Kaplan-Meier curves of progression free survival were analyzed using log-rank test. Cox-regression was employed for multivariate analysis. A p-value of < 0.05 was considered statistically significant.

## Results

### Impact of minimal extrathyroidal extension on the prevalence of metastases at initial diagnosis

#### Study cohort

One hundred sixty patients (80 matching partners) met the inclusion criteria for the assessment of M-ETE as a risk factor for metastatic disease at initial diagnosis. The mean patient age was comparable among groups (M-ETE: 48 years, range: 10–78 years; TCT: 45 years, range: 16–75 years).

#### Histopathology

PTC entails the most frequent entity representing 69/80 patients (86.3 %) each in the subcohort of patients with minimal extrathyroidal extension and in the subcohort of patients with tumors confined to the thyroid. FTC made up for 11 out of 80 patients (13.8 %) each.

#### Risk factor assessment

To account for possible confounders with regards to metastases at initial diagnosis in both groups we examined the following risk factors in patients with and without ETE and provided a summary in Table 1:

**Table 1:** Patient characteristics and assessment of statistically significant differences among both study cohorts with regards to clinical and histopathological risk factors as well post-primary treatment

Category		M-ETE	TCT	*p*-value
**Sex**	**Male, n (%)**	20 (25.0 %)	23 (28.7 %)	0.72
	**Female, n (%)**	60 (75.0 %)	57 (71.3 %)	
**Age**	**Mean (years)**	48.6 (10–78)	45.1 (16–75)	
	**> 55 years, n (%)**	24 (30.0 %)	20 (25.0 %)	0.60
**Histo-pathology**	**PTC, n (%)**	69 (86.3 %)	69 (86.3 %)	
	**FTC, n (%)**	11 (13.8 %)	11 (13.8)	
	**Tumor size (mm)**	22.1	21.9	0.93
	**Multifocal tumor, n (%)**	16 (20.0 %)	17 (21.3 %)	1.00
	**R1 resection, n (%)**	15 (18.8 %)	8 (10.0 %)	0.18
**Post-primary treatment**	**EBRT, n (%)**	13 (16.3 %)	1 (1.3 %)	0.001
	**Median cumulative**^**131**^**Iodine activity (GBq)**	10.0 (range: 0–31)	8.0 (range: 0–27)	< 0.001

*M-ETE* minimal extrathyroidal extension, *TCT* tumor confined to the thyroid, *PTC, FTC, EBRT* external beam radiation therapy, *GBq* Gigabecquerel

##### Tumor size

Mean tumor size in the entire study cohort was 22.0 mm and did not differ among groups (patients with M-ETE: 22.1 mm, patients with TCT: 21.9 mm; *p* = 0.93).

##### Male sex

Men make up for 23/80 patients (28.7 %) in the subgroup of patients with TCT and 20/80 patients with M-ETE (25.0 %). This difference was not statistically significant (*p* = 0.72).

##### Age > 55 years

The M-ETE cohort entailed 24/80 (30.0 %) patients > 55 years, the TCT cohort entailed 20/80 (25.0 %) patients > 55 years. This difference was not statistically significant (*p* = 0.60).

##### Multifocal tumor lesions

Multifocal tumor growth was distributed almost equally among both groups and was present in 16/80 (20.0 %) patients with M-ETE and 17/80 (21.3 %) patients with TCT (*p* = 1.0).

##### Incomplete tumor resection

In 23/160 patients (14.4 %) R1-resection was performed. Within the M-ETE cohort the proportion of patients with an initial R1 resection was slightly higher, but statistical significance was not reached (18.8 % vs. 10.0 %; *p* = 0.18).

##### Prior treatment

Patients in the M-ETE cohort had undergone EBRT at significantly higher rates (16.3 % vs. 1.3 %; *p* = 0.001) than their counterparts with TCT. Additionally, these patients had received higher radioactive iodine activities than their matching partners (median ^131^I activity: 10.0 GBq vs. 8.0 GBq; *p* = < 0.001).

#### Analysis of metastases at initial diagnosis

**Fig. 1:**
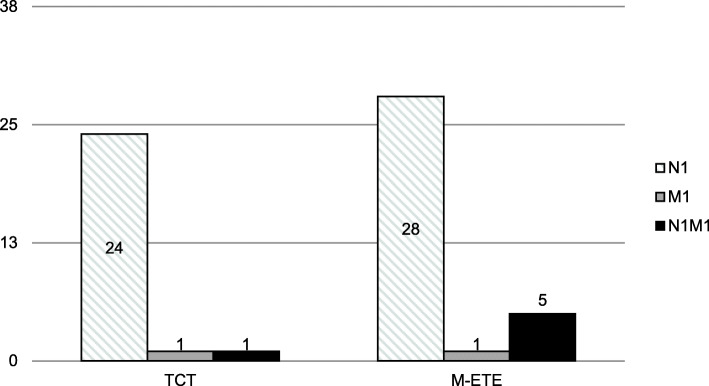
Bar charts showing the number of patients with nodal and remote metastases at initial diagnosis separately for patients with tumors showing minimal extrathyroidal extension and for patients with tumor confined to the thyroid gland. No statistically significant differences regarding the presence of initial N1- (*p* = 0.62) or M1-disease were observed among groups (*p* = 0.28). TCT: tumor confined to the thyroid gland; M-ETE: tumor showing minimal extrathyroidal extension

Metastases at initial diagnosis were observed more frequently in patients with M-ETE (34/80, 42.5 %) than patients with TCT (26/80, 32.5 %), but statistical significance was not reached (*p* = 0.25). 24/80 (30.0 %) of the TCT patients had N1M0 disease, 1/80 (1.3 %) N1M1 disease, and 1/80 (1.3 %) N0M1 disease. 28/80 (35.0 %) of the M-ETE patients had N1M0 disease, 1/80 (1.3 %) N0M1 disease, and 5/80 (6.3 %) had N1M1 disease. There were also no statistically significant differences in the prevalence of remote metastases (M-ETE: 7.5 %, TCT: 2.5 %; *p* = 0.28). Figure 1 provides an overview of the patients who presented with N1-, M1-, and N1M1-disease, respectively.

#### Recurrence rate

Recurrence was defined as the detection of recurring structural disease by any imaging modality. In the M-ETE group, recurrence was detected in 16/80 (20 %) of patients, whereas the recurrence rate was 20/80 (25 %) in the TCT group.

**Fig. 2:**
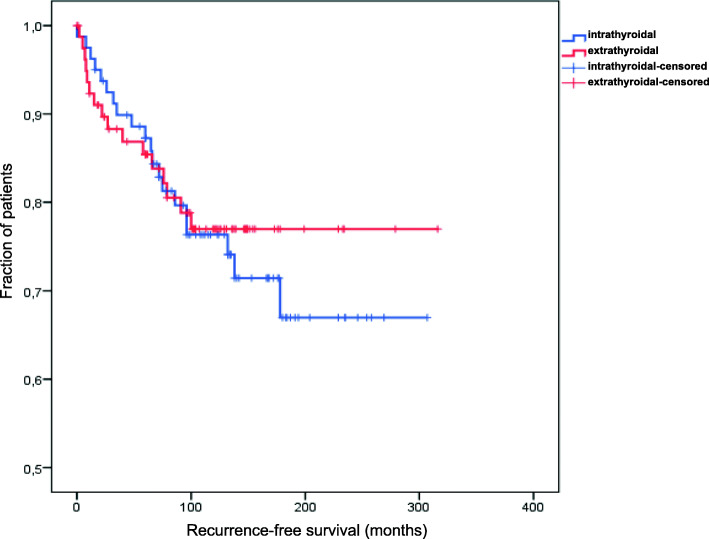
Kaplan-Meier curves showing the time to recurrence in months for patients with (red line) and without (blue line) M-ETE. No statistically significant differences were observed (*p* = 0.72)

**Fig. 3:**
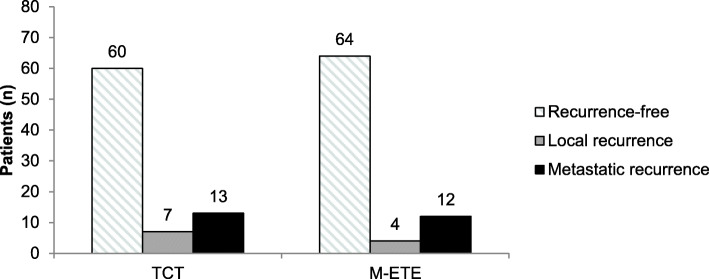
Bar charts showing the recurrence rate for patients with tumors showing minimal extrathyroidal extension and their matching partners with tumors confined to the thyroid gland. No statistically significant differences were observed for the rate of local (*p* = 0.53) and nodal (*p* = 1.0) recurrence. TCT: tumor confined to the thyroid gland; M-ETE: minimal extrathyroidal extension

Kaplan-Meier analysis did not reveal statistically significant differences among patients with TCT vs. patients with M-ETE with regards to recurrence (20/80, 25.0 % vs. 16/80, 20.0 %; *p* = 0.72; median recurrence-free survival not reached in both groups). Local recurrence occurred in 4/80 (5.0 %) patients with M-ETE and 7/80 (8.8 %) of patients with TCT (*p* = 0.53). Diagnosis of new lymph node or remote metastases was found in 12/80 (15.0 %) patients with M-ETE and 13/80 (16.3 %) patients with TCT (*p* = 1.0). Kaplan-Meier curves and box plots showing the time and localization of recurrence are provided in Figs. 2 and 3.

#### Multivariate analysis of the recurrence rate

Initial N1-disease (Odds Ratio: 2.70; *p* = 0.02) was a statistically significant risk factor for tumor recurrence. Minimal extrathyroidal extension was not associated with a higher recurrence rate (*p* = 0.17). None of the other assessed parameters were statistically significant risk factors for disease recurrence. An overview of assessed risk factors is provided in Table 2.

**Table 2:** Cox-regression model of risk factors for disease recurrence. *M-ETE *minimal extrathyroidal extension, *OR *Odds Ratio, *CI *confidential interval, *RAIT *radioactive iodine therapy, *EBRT *external beam radiation therapy

	*p*	Odds Ratio	95 % CI for Odds Ratio	
			CI lower	CI upper
M-ETE	0.17	0.56	0.25	1.28
Age > 55	0.27	1.65	0.68	3.99
Initial N1-disease	0.02	2.70	1.21	6.04
Initial M1-disease	0.08	3.28	0.87	12.31
R1-resection	0.93	1.05	0.36	3.05
RAIT activity	0.46	1.02	0.97	1.07
Multifocal disease	0.40	1.46	0.60	3.58
EBRT	0.76	1.22	0.35	4.29

## Discussion

In this study we were able to show that - even when accounting for other established risk factors - patients with M-ETE were not more likely to show metastases at initial diagnosis. Additionally, the rate of recurrence or development of metastases in the course did not differ significantly when compared to patients with a TCT.

However, post-primary treatment differed significantly among both cohorts, as M-ETE patients underwent RAIT with higher cumulative activities and EBRT at higher frequencies.

It is therefore still unanswered whether the lack of statistically significant differences regarding the recurrence rate is attributable to the differing treatment of both cohorts or the negligible impact of M-ETE on patient prognosis.

If the former is true, the diagnosis of M-ETE warrants a more extensive post-primary treatment. If the latter is true, M-ETE patients in this cohort were likely subject to overtreatment.

The answer to this question -and in a broader sense the role of adjuvant RAIT in general- is a matter of debate and currently hindered by a lack of evidence. It has to be answered conclusively by future randomized controlled trials focusing on patient-relevant outcome measures, as advised by the authors’ of the Martinique statements [21].

Similar to the present study, Furlan et al. did not observe statistically significant outcome differences between patients with M-ETE vs. TCT. Interestingly, patients diagnosed with FTC with TCT were more likely to show metastases at initial diagnosis [22] compared to their counterparts with FTC with M-ETE. A study by Shin et al. yielded comparable results, with no higher recurrence rate in patients with M-ETE vs. those without [23].

While the role of M-ETE has yet to be elucidated conclusively, the role of macroscopic extrathyroidal extension (ETE) appears to be clearer:

In 1991 a study by Akslen and Myking demonstrated that extrathyroidal extension (ETE) was a negative prognostic marker in a patient collective that is comparable to ours with regards to the distribution of age, sex, and histopathological subtype [24]. They separately assessed the prognostic impact of thyroid capsular invasion and major extrathyroidal growth, which they defined as any infiltration of skeletal muscle, large nerves or lipomatous tissue in accordance to the 1987 UICC classification. Both features were associated with a reduced survival time. In contrast to our study, a significant association between tumor capsular invasion and the presence of lymph node metastases was found. Interestingly, no statistically significant differences in survival times were found when comparing patients with thyroid capsular invasion vs. major extrathyroidal growth, which led the authors to the conclusion that tumor capsular infiltration should be viewed as a marker for early extrathyroidal extension.

This was confirmed by Yasumoto et al. in 1996, who analyzed different risk factors within a study cohort of 357 patients and showed that ETE defined any infiltration of the trachea, nerves or lymphoid tissue lead to an increase disease specific mortality rate [25]. Similar to our study, initial N1-disease was associated with a higher recurrence rate. Mean patient age and the fraction of FTCs were slightly higher than in our collective, whereas the sex distribution was comparable.

Studies examining the prognostic role of ETE have shown that patients with an infiltration into the soft-tissue posterior to the thyroid gland show a worse outcome than patients, in whom the soft-tissue anterior to the thyroid capsule is infiltrated. Still in both cases outcome is influenced negatively [26]. Yet in this analysis the extension of ETE is not taken into account, which leads to the question, whether any ETE is a bad prognostic marker or just macroscopic ETE.

To our knowledge this is the first study using the concept of matching partners comparing patients with similar tumor size and histopathology, with the only difference being the absence or presence of M-ETE.

Interestingly, age > 55 years was not significantly associated with a higher risk for disease. This is in contrast to a prior study by Trimboli et al. [27], who showed that high-risk patients (as stratified by ATA criteria [28]) older than 55 years had the highest risk for relapse and significantly shorter disease-free survival. A possible explanation is the assumably small number of high-risk patients, as patients with tumors showing macroscopic ETE were not included, and the generally smaller sample size. Additionally, age as an independent risk factor for tumor recurrence was tested on the entire study cohort, not separately for risk groups.

Limitations include that in our collective M-ETE patients were treated more aggressively, receiving EBRT at higher rates and receiving RAIT with higher activities of ^131^Iodine. This constitutes a potential confounder: Prior studies by Farahati et al. [29] as well as Tsang et al. [30] have shown lower recurrence rates in PTC with either residual disease or perithyroidal tumor infiltration, age > 40 years and nodal involvement undergoing additional EBRT. However, other studies have not replicated these results so that the benefit of adjuvant EBRT is questionable and thus the impact on our study results likely minor [8].

The intensified treatment in patients with M-ETE might also have been caused by the borderline significant higher prevalence of R1 resection in patients with M-ETE (18.8 % vs. 10.0 % in the TCT subgroup; *p* = 0.18):

All patients with R1 resection (14.4 %) underwent RAIT. Conversely, all patients, in whom RAIT was omitted, had had an R0 resection.

A limitation entails that histopathological reports were obtained from different institutes. As the interobserver variation in the assessment of minimal extrathyroidal extension is high, it is not out of question that some patients were misclassified, thus negatively impacting our study results [31]. Nonetheless, medical diagnostics and treatment do not always occur under hypothetical idealized conditions. Thus, our study results are, to a large extent, close to real-world clinical scenarios, in which DTC patients are referred with histopathological reports from different institutes and not exclusively from a reference pathologist for thyroid tumors.

Additionally, due to the follow-up time of 5 years, late recurrences could have been missed. However, the literature suggests that the majority of tumor recurrences in differentiated thyroid carcinoma occur within 5 years of initial diagnosis [32].

## Conclusions

A clear judgement with regards to the impact of M-ETE on patient outcome remains difficult. Prior studies have yielded conflicting results, which may partly be attributed to varying definitions and different degrees of extrathyroidal extension in the study subjects. Therefore, further trials and (as recently suggested [10]) a more standardized reporting system for the assessment of extrathyroidal extension seem warranted.

Still our results suggest that intensified treatment of patients with M-ETE might not be necessary; a diagnostic workup and therapy tailored to the individual risk profile that takes all known risk factors into account should be performed instead [28].

## Data Availability

The datasets generated and/or analyzed during the current study are not publicly available due to privacy legislation but are available from the corresponding author on reasonable request.

## Abbreviations

DTC: Differentiated thyroid cancer
M-ETE: Minimal extrathyroidal extension
PTC: Papillary thyroid carcinoma
FTC: Follicular thyroid carcinoma
TCT: Tumor confined to the thyroid gland
RAIT: Radioactive iodine therapy
EBRT: External beam radiation therapy
ETE: Extrathyroidal extension
